# Hydrometrocolpos With Polydactyly in Consanguineous Parents: A Case Report and Review of Literature

**DOI:** 10.7759/cureus.34880

**Published:** 2023-02-11

**Authors:** Touqa Khalil, Faaezuddin Syed, Basant Elaasser, Haroon A Javaid, Usama I Othman, Nabil Shehata

**Affiliations:** 1 Internal Medicine, Alfaisal University College of Medicine, Riyadh, SAU; 2 Radiology, Saudi German Hospital Group, Riyadh, SAU; 3 Pediatrics and Neonatology, Saudi German Hospital Group, Riyadh, SAU

**Keywords:** hydrometrocolpos, imperforate hymen, consanguinity, neonate, abdominopelvic mass, hydroureteronephrosis, post-axial polydactyly, neonatal hydrometrocolpos, abdominal cystic mass

## Abstract

Neonatal hydrometrocolpos (HMC) is a cystic dilatation of a neonate's vagina and uterus occurring secondary to congenital vaginal obstruction, with or without maternal estrogenic stimulation of uterine and cervical glands causing increased secretions during the prenatal and postnatal period. Diagnosis is made using ultrasonography and further confirmed by MRI. HMC in a neonate can rarely present with congenital anomalies such as polydactyly, which may indicate a variety of underlying genetic syndromes. There is a deficit in the literature as to whether the development of HMC in a neonate of consanguineous parents is an isolated finding or solely related to an underlying syndrome. We hope to help bridge this gap by reporting a case of a 12-day-old neonate presenting with hydrometrocolpos and polydactyly, born to consanguineous parents.

## Introduction

Neonatal hydrometrocolpos (HMC) is a cystic dilatation of the vagina and uterus occurring secondary to congenital vaginal obstruction. Most cases occur due to imperforate hymen, but other embryological pathologies such as vaginal atresia, transverse vaginal septum, and cloacal malformations can also lead to its development. Maternal estrogenic stimulation of uterine and cervical glands causing increased secretions during the prenatal and postnatal period has been shown to play a role [1]. Overall, it is a rare congenital anomaly that accounts for 15% of intra-abdominal cystic masses and has an incidence rate of 1 in 16,000 live births [2]. The presence of an intra-abdominopelvic cyst during the neonatal period should prompt pertinent investigations to be arranged in order to reach a diagnosis, provide symptomatic relief to the patient, and prevent associated complications. The diagnosis of HMC, either prenatally or postnatally, can be made using ultrasonography, which is further confirmed by MRI [3]. Antenatally, HMC can be visualized on ultrasound as a cystic mass present in the pelvic cavity with or without extension into the abdominal cavity. Moreover, additional findings of hydroureteronephrosis may also be seen due to ureteric compression and subsequent urinary backflow. Postnatally, HMC can manifest as an intraabdominal or vulvoperineal mass [4]. In some cases, individuals with HMC are completely symptom-free and present with complaints when they undergo puberty. This is attributed to the physiological activation of the hypothalamic-pituitary-gonadal axis, causing an increased rate of uterine secretions. Abnormal vaginal patency causes the secretions to accumulate in the vagina and ultimately to pool in the uterus, leading to its expansion and development of symptoms [5]. HMC in a neonate can rarely present with congenital anomalies such as polydactyly. The presence of both HMC and polydactyly may be indicative of a variety of underlying genetic syndromes such as McKusick-Kaufman syndrome (MKS), Bardet-Biedl syndrome (BBS), and Ellis-Van Creveld syndrome (EVC). The combination of congenital heart disease (CHD), postaxial polydactyly (PAP), and HMC characterize MKS. The diagnosis of MKS is based on the clinical diagnostic criteria, which requires the presence of HMC and PAP, with an absence of clinical or molecular genetic findings suggestive of an alternative diagnosis. BBS can mimic MKS as it has considerable clinical overlap with some additional age-dependent features such as obesity, retinal dystrophy, and intellectual disability. Hypogonadotropic hypogonadism and genitourinary malformations can also be seen in individuals born with BBS. Molecular genetic testing for both MKS and BBS reveals mutations in the same gene on chromosome 20p12 [6-8]. EVC is an ectodermal and chondral dysplasia characterized by polydactyly, short ribs, growth retardation, and heart defects. EVC and MKS, both recessively inherited disorders, share congenital heart defects and PAP. Ectodermal anomalies and osteochondrodysplasia distinguish EVC from MKS, which is associated with hydrometrocolpos. EVC can be detected prenatally using ultrasonography [8]. Here, we report a case of a 12-day-old neonate presenting with HMC and polydactyly, born to consanguineous parents, with a high possibility of an underlying genetic syndrome.

## Case presentation

**Figure 1:**
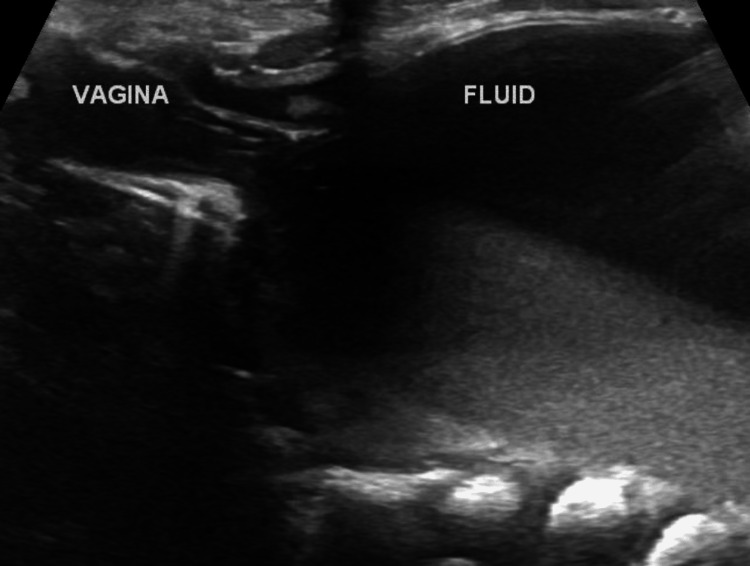
Ultrasound showing a pelviabdominal cyst composed of turbid fluid content arising from the vagina.

**Figure 2:**
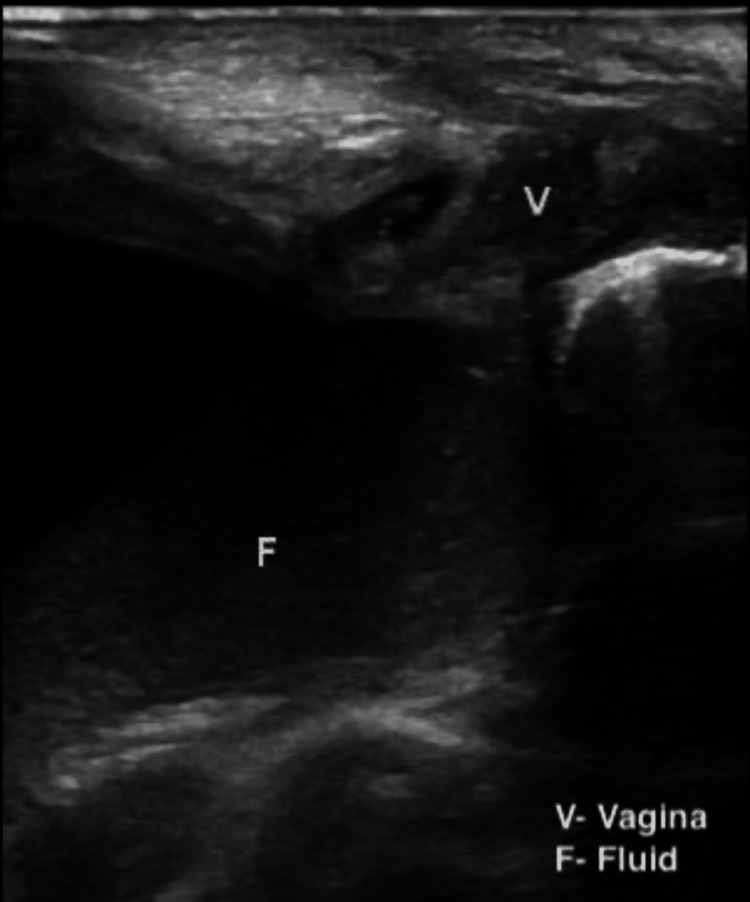
Ultrasound showing a pelviabdominal cyst composed of turbid fluid content arising from the vagina.

**Figure 3:**
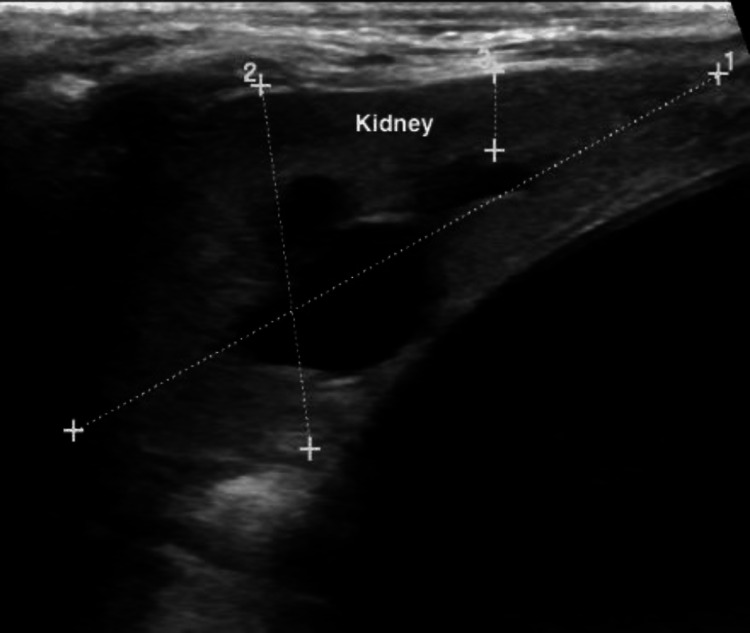
Ultrasound showing mild hydronephrosis.

**Figure 4:**
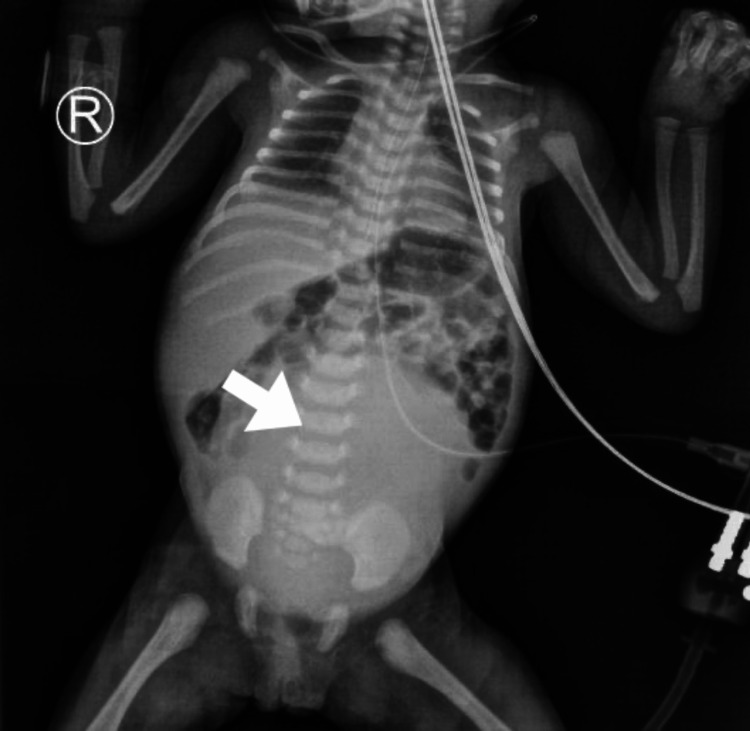
X-ray showing a large pelviabdominal cystic lesion shadow at the abdomen with no pathological calcifications.

**Figure 5:**
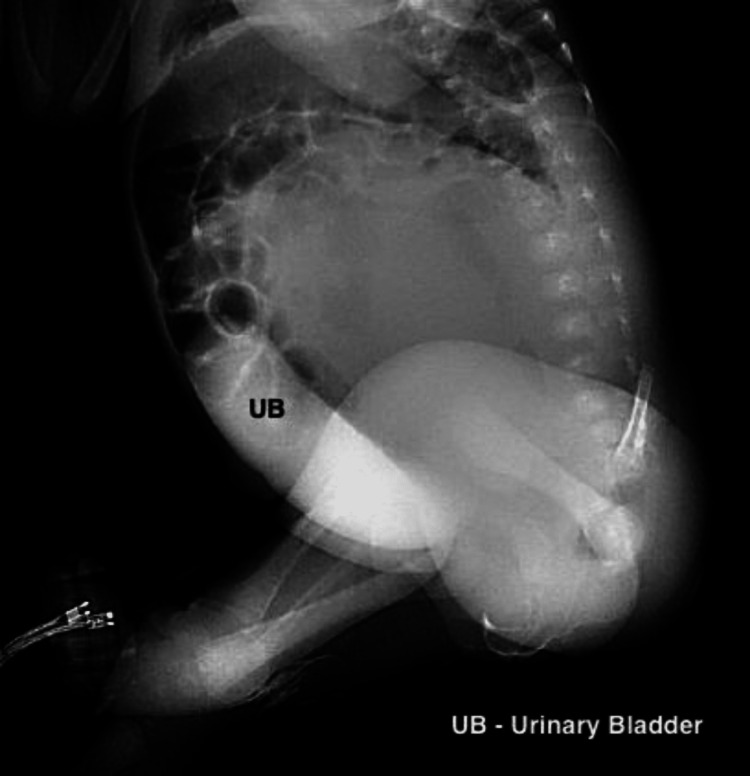
Cystourethrogram showing smooth filling of the urinary bladder.

**Figure 6:**
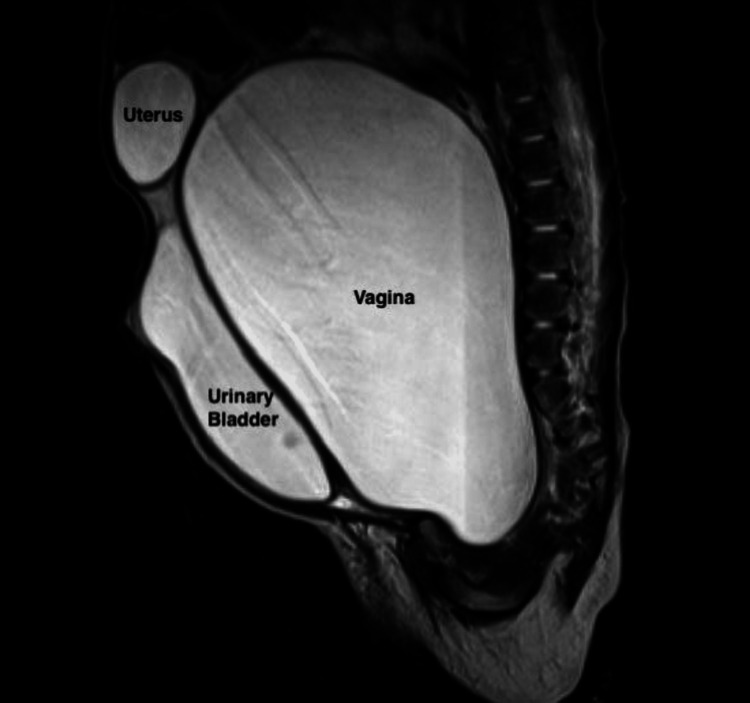
MRI in the sagittal plane shows the presence of a distended vagina containing fluid. The uterus is displaced superiorly, and the urinary bladder is compressed and pushed anteriorly.

**Figure 7:**
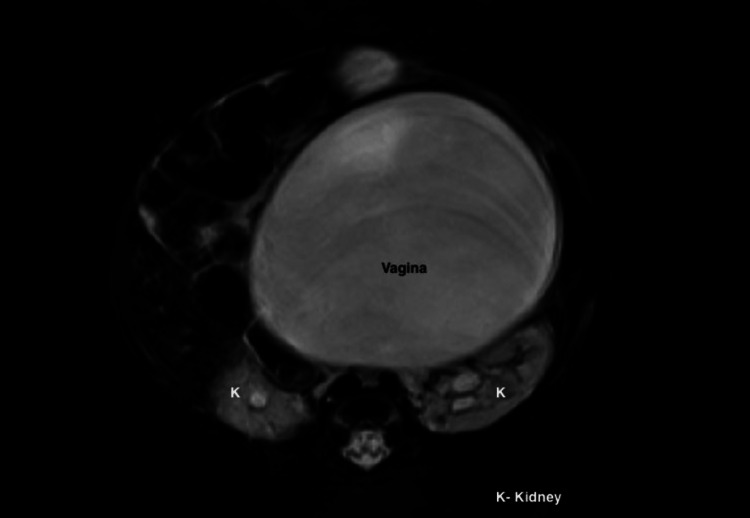
MRI in the axial plane showing a distended vagina containing fluid and bilateral hydronephrosis.

We present a 12-day-old baby girl, born preterm at 36 weeks of gestation, to a gravida 6, para 2, aborta 3 mother. Parental history was positive for consanguinity. Antenatal ultrasound revealed marked abdominal distention with bilateral hydronephrosis. She was born via spontaneous vaginal delivery with a cephalic presentation at a local hospital in northern Saudi Arabia. APGAR scores were eight and nine at one and five minutes, respectively. The growth parameters identified a birth weight of 2.5 kg, head circumference of 32 cm, and body length of 47 cm. The baby was referred to as a case of a huge pelvic cystic mass for advanced care at the neonatology department. Upon physical examination, the patient was vitally stable on room air with good activity and a pinkish appearance. She was able to tolerate oral food and had no signs of jaundice or dehydration. Normal eyes and ears, bilateral patent nose, and no evidence of cleft lip or palate were noted. Upper limbs were normal; however, an examination of the lower limb showed an extra digit in the right foot. Marked abdominal distension was appreciated, with a palpable mass extending from the suprapubic region to the epigastrium. Examination of the genitalia was positive for one common orifice for both the vagina and urethra. The patient had passed urine and stool upon admission. Nervous, cardiac, and respiratory examinations were otherwise insignificant. Initial lab investigations revealed several abnormalities (Table 1). Infectious serology and a metabolic screen for inborn errors of metabolism were unremarkable. Ultrasound of the abdomen and pelvis with doppler showed the presence of a large abdominopelvic cyst composed of turbid content measuring about 8 x 8 cm (Figures 1-2). The cyst was located posterior and inferior to the uterus, displacing the uterus superiorly and the urinary bladder anteriorly. The cyst compressed both ureters resulting in mild bilateral hydroureteronephrosis (Figure 3). Erect abdominal X-ray showed no evidence of pathological calcifications (Figure 4). An initial impression of a large hydrometrocolpos vs. ovarian cyst was made. Cyst urethrogram showed smooth filling of the urinary bladder with no mass or diverticular formation. There were no vesicovaginal fistulous tracts or signs of vesicoureteric reflux (Figure 5). MRI findings were significant for the presence of a distended vagina containing fluid with hemorrhagic products at various stages. Bilateral hydroureter and hydronephrosis were also present. A bicornuate uterus was noted, with findings suggestive of an imperforate hymen (Figures 6-7). 

**Table 1:** Initial laboratory findings.

Labs	Values
Total bilirubin	2.8 mg/dl (0.0-1.2)
Bilirubin Direct	0.70 mg/dl (0.0-0.5)
Calcium	12.10 mg/dl (9-11)
Phosphorous	2.25 mmol/L (0.74-1.52)
Potassium	6.5 mmol/L (3.7-5.9)
C-reactive protein (CRP)	0.8 mg/L (0.5)
Creatinine	1.13 mg/dl (0.57-1.11)

The patient was admitted to the neonatal intensive care unit (NICU) with the involvement of the following teams: pediatric surgery, pediatric urology, nephrology, genetics, and ophthalmology. She was maintained on incremental doses of oral feeding with IV fluids. Repeat blood tests showed irregularities in creatinine and potassium with values of 3.6 mg/dl (0.57-1.11) and 6.1 mmol/L (3.7-5.9), respectively. Decisions involving an urgent need for possible cystoscopy, vaginoscopy, midline laparotomy, vaginostomy, vaginoplasty, and vaginal pull-through were made by pediatric surgery and pediatric urology.

## Discussion

Hydrometrocolpos (HMC) is a rare malformation causing distention of the uterus and vagina due to the accumulation of secretions. These secreted products can either be urine or uterine mucus stimulated under the influence of maternal estrogen. A diagnosis of HMC is made when there is a vaginal outlet obstruction, such as imperforate hymen, vaginal atresia, etc., causing the buildup of secretions [5]. Depending on the severity of the case, HMC can have a wide variety of clinical manifestations. Neonatal HMC, in particular, poses a serious challenge for its diagnosis due to a wide spectrum of relative differentials. A pelvic mass extending to the abdomen can be due to neuroblastoma, ovarian cyst, intra-abdominal sacrococcygeal teratoma, mesoblastic nephroma, anterior cystic hygroma, bowel duplication, genitourinary anomalies, hydronephrosis, anterior sacral meningocele, or an intra-peritoneal fluid collection following organ failure [9]. Moreover, as with any abdominal mass, HMC can compress the surrounding structures leading to obstructive uropathy and constipation, or might even cause respiratory distress if large enough.

Ultrasound is the most widely used tool for diagnosing HMC, which normally reveals a cystic pelvic mass. MRI or CT scans can be used for definitive diagnosis in case of inconclusive results, the former being used in our case [10]. HMC is usually diagnosed either prenatally or during late childhood/early puberty. In our case, the HMC was diagnosed with suggestive antenatal findings of abdominal distention and bilateral hydronephrosis in the neonatal period. Therapeutic options vary as they mainly rely on the underlying etiology and extent of obstruction. They have ranged from correcting the underlying genital anomaly to surgical decompression in previously reported cases [4]. With imperforate hymen being the most common cause of HMC, a simple hymenectomy is often performed to relieve the obstruction [9]. However, in our case, even though the MRI showed suggestive findings of an imperforate hymen as the cause of HMC, a more invasive approach was planned. This decision was made due to the presence of bilateral hydroureteronephrosis seen on imaging caused by the enlarged HMC compressing the ureters. In addition to the incrementally increasing creatinine levels, this was indicative of a potential kidney injury. Such a scenario warranted an urgent decompressive approach for management.

HMC can be an isolated finding or, more commonly, associated with congenital anomalies such as polydactyly, as in our patient. This association is found in many syndromes, including MKS, BBS, and EVC. The presence of consanguinity in our patient's parents should be considered due to its association with autosomal recessive disorders. A study done in 2021 found the rate of consanguinity in Saudi Arabia to be 52%, 39.3% of which were first-cousin marriages [11]. Another study done in Qatar, where the consanguinity rates are comparable to Saudi Arabia (54%), showed that these marriages had a 2.9 increased risk of developing autosomal recessive disorders compared to the normal population. Even though the incidence of HMC is reported to be 0.006%, we anticipate a possibly higher incidence in Saudi Arabia due to a higher-than-average rate of consanguinity and autosomal recessive disorders [10,12].

The limitation of our case is the inability to perform molecular testing to confirm a possible underlying congenital syndrome despite having HMC in association with polydactyly. MKS, for instance, presents with cardiac anomalies; however, even with a confirmed cardiac defect on imaging, genetic analysis is required for diagnosis (chromosome 20p12 between D20S162 and D20S894 markers) [13]. Similarly, a diagnosis of BBS requires future surveillance by ophthalmologists to detect other commonly associated findings of this syndrome.

## Conclusions

HMC is a rare malformation that presents with an abdominopelvic cystic mass secondary to vaginal outlet obstruction. Diagnosis can be made pre- or postnatally using appropriate radiological modalities. One should have a high index of suspicion to allow immediate diagnosis and management to prevent any associated complications. There is a deficit in the literature as to whether the development of HMC in a neonate of consanguineous parents is an isolated finding or solely related to an underlying syndrome. Our case further elucidates the importance of bridging this gap to help with future in-depth studies.
